# Abnormal ECA-Binding Membrane Glycans and Galactosylated CAT and P4HB in Lesion Tissues as Potential Biomarkers for Hepatocellular Carcinoma Diagnosis

**DOI:** 10.3389/fonc.2022.855952

**Published:** 2022-03-22

**Authors:** Ying Kong, Hao Chen, Mengyu Chen, Yongshuai Li, Jiarong Li, Qi Liu, Huan Xiong, Tangxi Guo, Yan Xie, Yufeng Yuan, Xiao-Lian Zhang

**Affiliations:** ^1^ Hubei Province Key Laboratory of Allergy and Immunology, and Department of Immunology, Wuhan University School of Basic Medical Sciences, Wuhan, China; ^2^ Department of Pathology, Zhongnan Hospital of Wuhan University, Wuhan, China; ^3^ Department of Radiation and Medical Oncology, Zhongnan Hospital of Wuhan University, Wuhan, China; ^4^ Hepatobiliary and Pancreatic Surgery, Zhongnan Hospital of Wuhan University, Wuhan, China; ^5^ Allergy Department of Zhongnan Hospital, State Key Laboratory of Virology, Medical Research Institute Wuhan University School of Medicine, Wuhan, China

**Keywords:** lectin array, *Erythrina cristagalli* (ECA) lectin, hepatocellular carcinoma, catalase (CAT), prolyl 4-hydroxylase beta polypeptide (P4HB), membrane biomarker for diagnosis

## Abstract

Hepatocellular carcinoma (HCC) is one of the most common types of cancer. Despite decades of research efforts, the search for novel biomarkers is still urgently needed for the diagnosis of HCC and the improvement of clinical outcomes. Previous studies of HCC clinical biomarkers have usually focused on serum and urine samples (e.g., serum Alpha-fetoprotein (AFP). However, cellular membrane proteins in lesion tissues are less used in HCC diagnosis. The abnormal expression of membrane glycoproteins in tumor lesions are considered as potential targets for tumor diagnosis and tumor therapies. Here, a lectin array has been employed to screen and identify abnormal glycopatterns and cellular membrane glycans in HCC lesion tissues compared with adjacent non-tumor tissues. We found that there was significantly less expression of *Erythrina cristagalli* (ECA) lectin binding (Galβ1-3/β1-4) glycans on the cellular membrane of HCC lesion tissues compared with those of adjacent non-tumor tissues. Immunohistochemistry analysis further showed that ECA-binding ability on the membrane proteins of HCC tissues progressively decreased in different tumor-node-metastasis (TNM) stages (stage I to stage III) as the malignancy of liver cancer increased. Receiver operating curve (ROC) analysis showed ECA-binding ability yielding a sensitivity of 85% and specificity of 75%, and a combination of ECA and AFP has better clinical diagnostic efficiency, yielding a sensitivity of 90% and specificity of 85%, than ECA or AFP assay alone. ECA pull-down followed by mass spectrometry further showed that there was significantly less expression of ECA binding membrane catalase (CAT) and prolyl 4-hydroxylase beta polypeptide (P4HB) in HCC tissues compared with the adjacent non-tumor tissues. The abnormally increased expression of total CAT and P4HB and decreased expression of galactosylated membrane CAT and P4HB in HCC cell lines were correlated with an HCC metastasis status. Our findings suggest that abnormal declined ECA-binding galatosylated membrane glycans and two galactosylated-CAT and P4HB glycoproteins in lesion tissues are potential biomarkers in the diagnosis and/or metastasis prediction for HCC.

## Introduction

Hepatocellular carcinoma (HCC) is the most common type of primary liver cancer and the third leading cause of death among cancers worldwide according to World Health Organization—WHO 2020 (1, 2). The overall 5-year survival rates have been reported to be as low as about 18% around the world (3). The incidence and mortality of HCC have been increasing rapidly for the past several years worldwide, and this represents a considerable public health burden (4–7). The major risk factors of HCC include chronic infection with hepatitis B or C virus (HBV or HCV), type 2 diabetes, heavy alcohol intake, and obesity (8). The poor prognosis of HCC is due to the rapid progression and lack of specific symptoms of HCC and over 60% of patients are diagnosed at an advanced stage or when metastasis has occurred (9).

Several biomarkers have been identified for HCC, such as alpha-fetoprotein (AFP), apyrimidinic endodeoxyribonuclease 1 (APEX1), glypican 3 (GPC3), Dickkopf-1 (DKK1), and Golgi protein-73 (GP73) (10–14). Most clinical HCC biomarkers have usually focused on serum and urine samples (e.g., serum AFP). Among them, AFP is considered currently the most successful diagnostic marker for HCC. Measurement of serum abnormal AFP levels serves as a routine method in clinical HCC surveillance and diagnosis, whereas sensitivity and specificity are still poor (15). Despite decades of research efforts, the search for novel biomarkers is still urgently needed for the diagnosis of HCC and the improvement of clinical outcomes.

In general, membrane proteins play important roles in tumor cell survival and cell communication, as they function as transporters, receptors, anchors, and enzymes. They also served as potential targets for drugs that block receptors or inhibit enzymes related to diseases, and targets for tumor diagnosis and tumor immunotherapies (such as chimeric antigen receptor T cell (CAR-T)). However, to date, membrane proteins are rarely used for HCC diagnosis (16).

Over 50% of proteins in mammal cells and 70–90% of mammal cell membrane proteins and secreted proteins are N-glycosylated (17). Thus, we intended to explore glycoconjugates biomarkers on the cellular surface of human HCC and further assess their value and performance in the diagnosis of HCC.

Lectins are a group of proteins that have a significant carbohydrate-binding ability that could specifically recognize glycan structures on glycoproteins (18). Currently, lectin array and lectin-agarose are usually used for screening and enrichment of low abundant glycoproteins to discover new biomarkers for cancer diagnosis and therapy (19).

In this study, we extracted cell membrane proteins from HCC and adjacent tissues samples and performed lectin array with 35 kinds of different lectins, combined with mass spectrometry (MS), to identify membrane glycoproteins biomarkers for HCC diagnosis. We found significantly decreased *Erythrina cristagalli* lectin (ECA) lectin-binding ability and ECA-binding membrane glycoproteins catalase (CAT) and prolyl 4-hydroxylase beta polypeptide (P4HB) from HCC tissues samples as potential candidates with diagnostic and/or metastasis prediction value for human HCC. Our findings also provide a set of potential targets for HCC diagnostic application and therapeutic strategies.

## Materials and Methods

### Ethics Statements

The collection and use of all human HCC samples for research presented here were approved by the Ethical Committee of Wuhan University School of Medicine in Wuhan, China. Informed consent was obtained from each patient for the collection of the HCC samples. The study methodologies were conducted in accordance with the ethical guidelines of the Declaration of Helsinki.

### HCC Tissue Specimens

Surgically resected primary HCC tissues and paired adjacent non-tumor tissues were collected from a total of 41 HCC patients who had not experienced prior chemotherapy or radiotherapy at the Zhongnan Hospital of Wuhan University School of Medicine (Wuhan, China). Medical records were reviewed by study physicians to confirm the diagnosis of HCC and to record patient characteristics [e.g., AFP, tumor size, tumor number, tumor, node, and tumor-node-metastasis (TNM) classification]. A lectin array assay was performed using 10 HCC tissues and paired adjacent non-tumor tissues. Immunofluorescence was performed using 7 HCC tissues and paired adjacent non-tumor tissues. Immunohistochemistry was performed using 5 HCC tissues (TNM = I), 10 HCC tissues (TNM = II), and 5 HCC tissues (TNM = III). ECA pull down assay was performed followed by an LC–MS/MS or Western blot analysis using 4 HCC tissues and paired adjacent non-tumor tissues.

### Cell Lines

Human low metastatic liver cell MHCC-97L was a gift from Prof. Fubing Wang from the Hubei Key Laboratory of Tumor Biological Behaviors of the Zhongnan Hospital of Wuhan University School of Medicine, and high metastatic liver cell HCC-LM3 was purchased from the China Center for Type Culture Collection (CCTCC) of Wuhan University, China. Human hepatocellular carcinoma cells Huh7.5.1, normal liver cells L02, MHCC-97L, and HCC-LM3 were maintained in Dulbecco’s modified Eagle’s medium (DMEM) supplemented with 10% fetal bovine serum (FBS, Gibco) (Invitrogen, USA) at 37°C in a 5% CO_2_ atmosphere as previously described (20, 21).

### Membrane Protein Extraction

Membrane protein extraction was performed using a Membrane and Cytosol Protein Extraction Kit (Beyotime, Shanghai, China) (22). Briefly, 30–50 mg tissues or 3 × 10^6^ cells were resuspended in 1 ml of membrane protein isolation solution A and homogenized on ice (for tissue samples) or lysed by two freeze-thaw cycles at liquid nitrogen and 37°C water bath respectively (for cell samples). Cell debris was discarded after centrifugation at 700*g* at 4°C for 10 min. The supernatant was centrifuged at 14,000*g* at 4°C for 30 min to settle the membrane protein debris. The pellet was resuspended in 300 μl solution B and vortexed at a high speed for 5 s twice. After centrifugation at 14,000*g* at 4°C for 5 min, the resulting supernatant was collected as the membrane protein fraction.

### Lectin Array Analysis

Lectin array analysis was performed as previously described (20). In brief, 35 kinds of commercially available lectins from the Vector Laboratories (Burlingame, CA) and the Sigma-Aldrich were immobilized onto a microplate. The total membrane proteins (2 mg) from HCC tissues and adjacent non-tumor tissues were labeled with fluorescent dye Cy3 (GE Healthcare; Buckinghamshire, UK) and about 10–15 μg protein was spotted for each individual spot on the lectin microplate. The Mean Fluorescence Intensity (MFI) at 570 nm was determined on a SpectraMax^®^ i3x microplate reader (Molecular Devices, Sunnyvale, CA), and average backgrounds were removed. The lectin microarray data were normalized, and the fold change was evaluated by comparison of the data from HCC tissues with adjacent non-tumor tissues and then analyzed by using GraphPad Prism 9.0.

### Immunohistochemistry (IHC) and Image Analysis

The IHC staining was performed as follows. Briefly, the tissue sections were formalin-fixed and paraffin-embedded. Approximately 4 μm of tissue sections were deparaffinized, rehydrated, and subjected to antigen retrieval in boiling citrate buffer (Servicebio) containing 0.05% Tween 20 for 30 min, then blocked with 0.3% peroxide for 10 min and 5% bovine serum albumin (BSA) for 30 min. The sections were incubated with biotin-conjugated ECA (1:250; Vector) overnight at 4°C, and then were incubated with 1:10,000 dilution of horseradish peroxidase (HRP)-conjugated streptavidin at 37°C for 45 min according to the instructions of the manufacturer. Finally, tissue sections were incubated with 3′, 3′-diaminobenzidine (DAB) (Sigma) until a brown color developed, and was counterstained with Harris’ modified hematoxylin. The slides were scanned using the Phenoptics™ Vectra 3 System (Akoya Biosciences, Inc., USA), and the digital images were acquired at ×200 magnification using the Phenochart 1.0.2 software. The entire area of the slides was scored and quantified using inForm^®^ 2.4.0 Advanced Image Analysis software. Using supervised machine learning algorithms, images were scored in the mode of 0–3+(4-bin) which was divided into 3 bins as 0/1+, 1+/2+, 2+/3+ after the process of cell segmentation (detecting cells and their nuclear and membranous compartments) (23). Percentage positivity of the cell nuclei and membrane within each bin was presented and the H-score is calculated using the percentages in each bin and range from 0 to 300. The sensitivity and specificity calculations were performed as described in the previous study.

### Immunofluorescence (IF) Analysis

The IF staining was performed as follows: 8 μm of frozen sections was set from −80°C to room temperature for 15 min and fixed with 4% paraformaldehyde for 10 min. After washing with 1× PBS, the cells were then incubated with 5% BSA for 30 min at room temperature and with biotin-conjugated ECA (1:25; Vector) overnight at 4°C. After washing with 1× PBS, the cells were incubated with FITC-conjugated streptavidin (1:500; EY) and 4′,6-diamidino-2-phenylindole (DAPI) (1:5,000; Sigma-Aldrich) in the dark for 30 min at room temperature. The coverslips were washed with PBS, mounted, and analyzed with a Leica Aperio VERSA 8 microscope (Leica Biosystems Richmond, Inc., USA).

### ECA Lectin Pull-Down Assay

ECA pull-down assay was performed as follows: biotin-ECA was mixed with total membrane proteins to the final concentration of 20 μg/ml and incubated at 4°C overnight. Then 30 μl streptavidin agarose resin (Thermo Scientific, USA) was washed with 1× PBS three times, resuspended with the protein mixture, and incubated at 4°C overnight. After being washed with 1× PBS three times to remove the unbound proteins, the resin was boiled at 100°C for 10 min and followed by SDS-PAGE and western blot analysis. The total membrane protein concentration was measured using a BCA protein assay kit (Fermentas, USA).

### TripleTOF/TOF-Mass Spectrum (MS) Analysis

In brief, 25 μl of membrane proteins extracted from HCC tissue and paired adjacent non-tumor tissue were prepared by biotin-conjugated ECA plus streptavidin agarose resin pull down, and then analyzed with SDS-PAGE and stained by commassie blue staining solution. The corresponding protein bonds were excised for MS analysis by the Wuhan Institute of Biotechnology of China. For protein identification, MS/MS spectra acquired by TripleTOF 5600+ were searched with ProteinPilot v.4.5 against the Uniprot-SwissProt human reference proteome database, using the Paragon Algorithm. The parameters were set as below: Sample Type, Identification; Cys Alkylation, Iodoacetamide; Digestion, Trypsin; Search Effort, Rapid ID. Only proteins with a threshold >95% confidence (>1.3 Unused Score) were considered for protein identification.

### Western Blot

Approximately 10 μg of total membrane and ECA binding membrane proteins by ECA lectin pull-down assay were subjected to 10% SDS-PAGE. After electrophoresis, the gels were transferred onto PVDF membranes (Millipore, Germany) and the membranes were blocked with 5% skim milk at room temperature for 2 h. The blot was probed with rabbit polyclonal antibody against CAT and P4HB (1:1,000; ABclonal) as primary antibodies at 4°C overnight, respectively, and incubated with goat anti-rabbit antibody IgG as a secondary antibody (1:10,000; Proteintech) at 37°C for 45 min, and developed using an ECL system (UVP Bioimaging, USA). Na^+^/K^+^-ATPase (ABclonal, Wuhan, China) was used as an internal membrane control for quantitation, and densitometric analysis of each band was measured using Image J software.

### Statistical Analysis

Data were presented as mean ± SD. Differences between the two groups were tested by unpaired Student’s t tests. Differences between more than two groups were tested by one-way ANOVA followed by Sidak’s multiple comparisons test. GraphPad Prism software (Version 9.0) was used to determine statistical significance. *P*-values under 0.05 were considered statistically significant (*p <0.05, **p <0.01, *** *p <*0.001, ****p <0.0001). NS represents no statistical significance.

## Results

### Significantly Declined ECA Binding Membrane Glycans of HCC Lesion Tissues Compared to Adjacent Non-Tumor Tissues With Lectin Array

**Figure 1:**
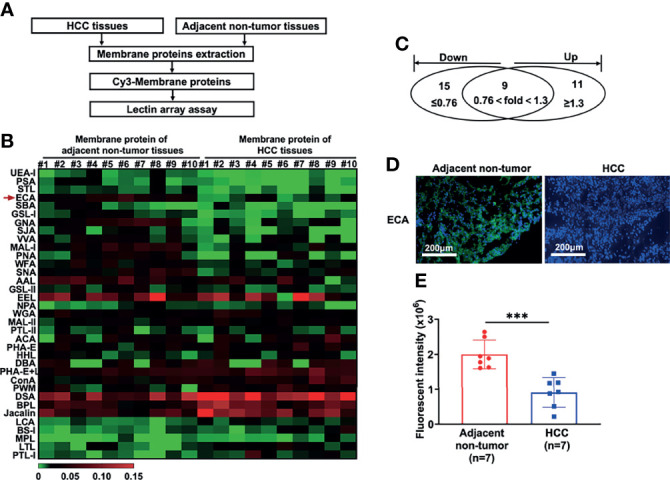
Lectin array and immunofluorescence analysis of glycopatterns on membrane proteins of HCC tissues and adjacent non-tumor tissues. **(A)** Workflow of the strategy for the determination of differentially expressed membrane glycopatterns in HCC tissues compared with adjacent non-tumor tissues. **(B, C)** Expressions of glycans on the membrane proteins between HCC tissues and adjacent non-tumor tissues analyzed by 35 different lectins presented as a heatmap **(B)** and Venn diagram **(C)**. #1 to #10: 10 HCC patients; **(D, E)** Immunofluorescence analysis of fluorescence intensity of glycans binding with ECA on cell membrane proteins between HCC and adjacent non-tumor tissues. Images are stained as FITC-conjugated ECA (green, membrane) and DAPI (blue, nuclei). Fluorescent intensities in **(E)** are presented as the mean ± SD using unpaired Student’s t-tests (***p < 0.001).

HCC tissues and paired adjacent non-tumor tissues were collected as described in *Materials and Methods* (**Table 1**). To identify the differentially expressed cellular surface glycoconjugates of HCC tissues compared to adjacent non-tumor tissues, a lectin microarray analysis with 35 kinds of different lectins was performed on 10 pairs of purified membrane proteins from 10 HCC and adjacent non-tumor tissues and analyzed with heatmap by Graphpad Prism 9.0 (**Figures 1A, B**). A total of 35 kinds of lectins and their corresponding binding glycans are shown in **Table 2**. We observed differential expression of glycopatterns of membrane proteins in HCC lesion tissues compared with adjacent non-tumor tissues **(** **Table 3** **)**. A 1.3-fold change (FC) cut-off was applied to classify glycans binding with lectins as up (≥1.3-fold) or down-regulated (≤0.76-fold). A total of 9 lectins that could bind with cellular membrane glycans of HCC tissues showed up-expressed MFI with 1.3 times (**Table 3**) and a total of 15 lectins showed down-expressed MFI with 0.76 times over adjacent non-tumor tissues (**Table 3** and **Figure 1C**). Among downregulated glycan-binding lectins, 11 lectins showed statistical significance and it is noticeable that ECA was presented the most significant difference (FC = 0.307, ****p <0.0001) (**Table 3**) and was selected to further confirm its association with HCC. Because Galβ1-3GlcNAc/Galβ1-4GlcNAc are usually considered as binding ligands for ECA (25), we postulate that abnormal declined ECA-binding membrane galactosylated glycans expression in lesion tissues of HCC samples compared with adjacent non-tumor tissues.

**Table 1:** Summary of clinical characteristics of human HCC samples.

Groups	For lectin array analysis	For IF analysis	For MS analysis	For IHC analysis	For WB analysis
Cases (24)	10	7	1	20	3
Gender					
Male	8	6	1	18	2
Female	2	1	–	2	1
Age (year)					
<50	5	1	–	2	–
≥50	5	6	1	18	3
AFP (ng/ml)					
<20	4	2	1	12	2
≥20	6	5	–	8	1
HBV-DNA (copies/ml)					
<1.00E+03	8	5	1	16	3
≥1.00E+03	2	2	–	4	–
Tumor size					
<5	5	3	1	9	3
≥5	5	4	–	11	–
Tumor number					
Single	9	7	1	16	3
Multiple	1	0	–	4	–
Vascular invasion					
Absent	9	2	1	16	1
Present	1	5	–	4	2
Metastasis					
Absent	8	5	1	11	3
Present	2	2	–	9	–
TNM stage					
Stage I	4	0	–	5	1
Stage II	4	4	1	10	2
Stage III	2	3	–	5	–

**Table 2:** Different glycan patterns between HCC tissues and adjacent non-tumor tissues by lectin microarray analysis.

No.	Abbr.	Source	Preferred carbohydrate specificity	Company
1	AAL	*Aleuria aurantia*	Fucα1-6GlcNAc, Fucα1-3(Galβ1-4)GlcNAc	Vector (USA)
2	ACA	*Amaranthus caudatus*	Galβ1-3GalNAc	Vector (USA)
3	BPL	*Bauhinia purpurea*	Galβ1-3GalNAc, GalNAc	Vector (USA)
4	BS-I	*Bandeiraea simplicifolia*	α-Gal, α-GalNAc, Galα-1,3Gal, Galα-1,6Glc	Sigma-Aldrich (USA)
5	ConA	*Canavalia ensiformis*	6(Manα1-3)Man, terminal GlcNAc	Calbiochem
6	DBA	*Dolichos biflorus*	αGalNAc	Vector (USA)
7	DSA	*Datura stramonium*	(GlcNAcβ1-4)n, Galβ1-4GlcNAc	Vector (USA)
8	ECA	*Erythrina cristagalli*	Galβ1-3GlcNAc, Galβ1-4GlcNAc	Vector (USA)
9	EEL	*Euonymus europaeus*	Galα3Gal	Vector (USA)
10	GNA	*Galanthus nivalis*	High-Mannose,Manα1-3Man	Vector (USA)
11	GSL-I	*Griffonia simplicifolia*	αMan	Vector (USA)
12	GSL-II	*Griffonia simplicifolia*	Agalactosylated Tri/tetra-antennary glycans	Vector (USA)
13	HHL	*Hippeastrum hybrid*	High-Man, Manα1-3Man, Manα1-6Man	Vector (USA)
14	Jacalin	*Artocapus integrifolia*	Galβ1-3GalNAcα-Ser/Thr(T)	Vector (USA)
15	LCA	*Lens culinaris*	Fucα1-6GlcNAc, α-D-Glc, α-D-Man	Vector (USA)
16	LTL	*Lotus tetragonolobus*	Fucα1-3Galβ1-4GlcNAc	Vector (USA)
17	MAL-I	*Maackia amurensis*	Galβ1-4GlcNAc	Vector (USA)
18	MAL-II	*Maackia amurensis*	Siaα2-3Gal	Vector (USA)
19	MPL	*Maclura pomifera*	Galβ3GalNAc	Vector (USA)
20	NPA	*Narcissus pseudonarcissus*	High-Man, Manα1-6Man	Vector (USA)
21	PHA-E	*Phaseolus vulgaris*	Bisecting GlcNAc	Vector (USA)
22	PHA-E+L	*Phaseolus vulgaris*	Bisecting GlcNAc, bi-antennary	Vector (USA)
			N-glycans	
23	PNA	*Peanut*	Galβ1-3GalNAcα-Ser/Thr(T)	Vector (USA)
24	PSA	*Pisum sativum*	core-fucosylated, trimannosyl structure	Sigma-Aldrich (USA)
25	PTL-I	*Psophocarpus tetragonolobus*	GalNAc	Vector (USA)
26	PTL-II	*Psophocarpus tetragonolobus*	Gal	Vector (USA)
27	PWM	*Sambucus nigra*	N−acetyl−D−glucosamine	Vector (USA)
28	SBA	*Solanum tuberosum*	α or β GalNAc, GalNAcα1-3GalNAc	Sigma-Aldrich (USA)
29	SJA	*Sophora japonica*	βGalNAc	Vector (USA)
30	SNA	*Soybean*	Sia2-6Gal/GalNAc	Vector (USA)
31	STL	*Solanum tuberosum*	GlcNAcβ1-4GlcNAc, Mixture Man5 to Man9	Vector (USA)
32	UEA-I	*Ulex europaeus*	Fucα1-2Galβ1-4GlcNAc	Sigma-Aldrich (USA)
33	VVA	*Vicia villosa*	GalNAcα1-3Gal	Vector (USA)
34	WFA	*Wisteria floribunda*	GalNAcβ1-4GlcNAc, Galβ1-3(-6)GalNAc	Vector (USA)
35	WGA	*Triticum vulgaris*	GlcNAc	Sigma-Aldrich (USA)

**Table 3:** Lectin binding glycopatterns of membrane glycoproteins between HCC tissues and adjacent non-tumor tissues.

Lectin	Carbohydrate specificity	Fold change (HCC/Adjacent non-tumor)
UEA-I	Fucα1-2Galβ1-4Glc(NAc)	0.147**
PSA	core-fucosylated, trimannosyl structure	0.225**
STL	GlcNAcβ1-4GlcNAc, Mixture Man5 to Man9	–
ECA	Galβ1-4GlcNAc, Galβ1-3GlcNAc	0.307****
SBA	α or β GalNAc, GalNAcα1-3GalNAc	0.372*
GSL-I	αMan	0.394**
GNA	High-Mannose,Manα1-3Man	0.412**
SJA	βGalNAc	0.428*
VVA	GalNAcα1-3Gal	0.514*
MAL-I	Galβ-1,4GlcNAc	0.523**
PNA	Galβ1-3GalNAcα-Ser/Thr(T)	–
WFA	GalNAcβ1-4GlcNAc, Galβ1-3(-6)GalNAc	0.636*
SNA	Sia2-6Gal/GalNAc	0.660*
AAL	Fucα1-6GlcNAc, Fucα1-3(Galβ1-4)GlcNAc	–
GSL-II	Agalactosylated Tri/tetra-antennary glycans	–
EEL	Galα3Gal	–
NPA	High-Man, Manα1-6Man	–
WGA	GlcNAc	–
MAL-II	Siaα2-3Gal	–
PTL-II	Gal	–
ACA	Galβ1-3GalNAc	–
PHA-E	Bisecting GlcNAc	–
HHL	High-Man, Manα1-3Man, Manα1-6Man	–
PHA-E+L	Bisecting GlcNAc, bi-antennary N-glycans	–
DBA	αGalNAc	1.330*
ConA	6(Manα1-3)Man, terminal GlcNAc	1.522*
PWM	N−acetyl−D−glucosamine	1.685*
DSA	(GlcNAcβ1-4)n, Galβ1-4GlcNAc	1.769**
BPL	Galβ1-3GalNAc, GalNAc	1.813**
Jacalin	Galβ1-3GalNAcα-Ser/Thr(T)	1.830*
LCA	Fucα1-6GlcNAc, α-D-Glc, α-D-Man	1.881***
MPL	Galβ1-3GalNAc, GalNAc	–
BS-I	α-Gal, α-GalNAc, Galα-1,3Gal, Galα-1,6Glc	–
LTL	Fucα1-3Galβ1-4GlcNAc	2.490***
PTL-I	Galβ1-3GalNAc, GalNAc	–

–: no significance; *p ＜ 0.05; **p < 0.01; ***p < 0.001; ****p < 0.0001.

We then performed immunofluorescence (IF) analysis using FITC-conjugated-ECA staining with 7 paired HCC tissues and adjacent non-tumor tissues to confirm the galactosylated glycan expression profiles. Consistent with the lectin array analysis, HCC tissues presented dramatically decreased green FITC fluorescence of ECA compared to adjacent non-tumor tissues (**Figures 1D, E**).

### Significantly Declined ECA Binding-Glycans in Different TNM Stage of HCC Lesion Tissues

**Figure 2:**
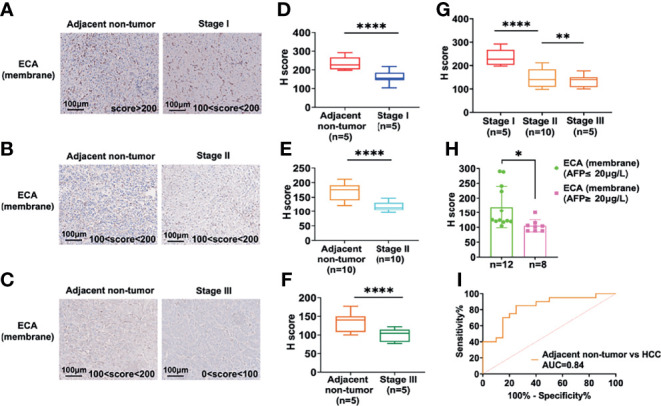
IHC analysis of the ECA-binding galactosylated glycans in the different stages of HCC and adjacent non-tumor tissues. **(A–F)** IHC analysis of staining intensity of glycans binding with ECA on cell membrane proteins among HCC tissues and adjacent non-tumor tissues. Images are stained as brown (HRP-avidin plus biotin-conjugated ECA) and blue (hematoxylin, nuclei). **(A–C)** are representative data for **(D–F)**. **(D, E)** are statistical analysis for **(A–C)**, respectively. **(G)** Comparison of staining intensity of ECA between different stages of HCC tissues. **(H)** Correlation analysis of serum AFP levels and ECA binding affinity. **(I)** ROC curves analysis of ECA between HCC tissues and adjacent non-tumor tissues. Significance analysis for **(D–F)** and **(H)** was performed using unpaired Student’s t-tests, and for G with one way ANOVA followed by Sidak’s multiple comparisons test (*p < 0.05, **p < 0.01; ****p < 0.0001).

Twenty HCC tissue samples (TNM I = 5, TNM II = 10, TNM III = 5) with paired adjacent non-tumor tissues served to confirm the expression change and evaluate the potential clinical significance of ECA using immunohistochemistry (IHC) (**Table 4**). The staining intensity of ECA showed a significantly lower level in HCC tissues of different TNM stages (from Stage 1 to Stage III) compared with those in adjacent non-tumor tissues (****p <0.0001) (**Figures 2A–F**). The binding affinity of ECA to cellular membrane glycans of HCC decreased with significant progressive from early Stage I to late Stage III (Stage I *vs* late Stage II: ****p <0.0001, Stage I *vs* late Stage III: ****p <0.0001, Stage II *vs* Stage III: ***p = 0.0010, **Figure 2G**). The IHC analysis result is positively correlated with the lectin blot data. These results indicated that decreased β1, 3/β1,4 galactosylated glycans binding with ECA may be related to the malignant progression and differentiation of HCC. Further, an ROC curve analysis was used to test the performance of ECA for HCC diagnosis (**Figure 2H**). The downregulation of ECA showed good performance in distinguishing HCC tissues from adjacent non-tumor tissues control, and the area under the curve (AUC) values are 0.84 (95% confidence interval—CI: 0.7084 to 0.9616), with a sensitivity of 85% (95% CI = 63.96–94.76%) and specificity of 75% (95% CI = 53.13–88.81%).

**Table 4:** Immunohistochemistry analysis of ECA in different TNM stages of HCC and adjacent non-tumor tissues.

TNM Stage		N	Scores for ECA			
			Low	Moderate		High
I	HCC	5	0		5	0
	Adjacent	5	0		1	4
II	HCC	10	0		10	0
	Adjacent	10	0		7	3
III	HCC	5	2		3	0
	Adjacent	5	0		5	0

Membrane staining was scored according to four categories: 0 for ‘no staining’, 1+ for ‘light staining visible only at high magnification’, 2+ for ‘intermediate staining’ and 3+ for ‘dark staining of linear membrane. Scores = 1 × (% of 1+ cells) + 2 × (% of 2+ cells) + 3 × (% of 3+ cells) (23).

Low: score ＜100; Moderate: 100＜score＜200; High: score ＞200.

Then we further analyzed the correlation between HCC serum AFP concentrations and binding affinities of lectin ECA to membrane proteins of HCC tissues. The subjects were divided into two groups according to the serum AFP concentration cut-off value 20 ng/L, as shown in **Figure 2I**. The high concentration AFP group (≥20 ng/L) showed a significantly lower ECA IHC score compared with the low concentration AFP group (≤20 ng/L), which indicates that the binding affinity of lectin ECA with HCC membrane glycoproteins is negatively correlated with HCC serum AFP concentrations.

We further combined decreased ECA with increased AFP to access whether this combination could enhance the diagnostic performance. The results showed that the combination of ECA and AFP results yielded a better clinical diagnostic efficiency between HCC tissues and adjacent non-tumor tissues than ECA or AFP assay alone, and yielded a sensitivity of 90% and specificity of 85%, while the sensitivity of AFP used alone was only 40%. These results imply that declined β1,3/β1,4 galactosylated glycans-binding ECA may be a potential biomarker for HCC diagnosis.

### Identification of Significantly Declined ECA-Binding Membrane Galactosylated CAT and P4HB Protein Expression in HCC Tissues

**Figure 3:**
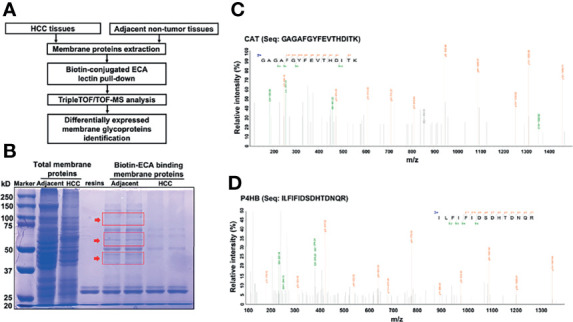
Identification of significantly decreased membrane glycoprotein galactosylated CAT and P4HB in HCC tissues with LC-MS/MS. **(A)** Workflow showing the strategy for the identification of decreased membrane galactosylated CAT and P4HB glycoproteins in HCC tissues. **(B)** SDS-PAGE analysis of the differential membrane proteins between adjacent non-tumor tissues and HCC tissues pull downed by biotin-conjugated ECA. The differentially expressed membrane protein bands indicated by red arrow were excised for MS analysis. **(C)** The amino-acid sequence (GAGAFGYFEVTHDITK) from MS is identified as CAT. **(D)** The amino-acid sequence (ILFIFIDSDHTDNQR) from MS is identified as P4HB.

Based on the above results, we then sought to identify specific membrane galactosylated glycoproteins in HCC. For protein identification, we employed the workflow as summarized in **Figure 3A**. ECA-binding membrane proteins from HCC tissues and adjacent non-tumor tissues were pulled down by biotin-ECA plus streptavidin-resin pull-down assay, followed by SDS-PAGE and coomassie blue staining shown in **Figure 3B**. The differentially expressed protein bands (indicated by the red arrow) between HCC tissue and adjacent non-tumor tissue were excised and analyzed by liquid chromatography-tandem mass spectrometry (LC–MS/MS). A total of 169 differentially expressed proteins were identified, among which 69 proteins were consistent with the molecular weights (MWs) of corresponding excised membrane proteins. The protein list is filtered based on Unused ProtScore, from our MS results (**Table 5**). Both CAT and P4HB are displayed as the two highest coverage and scored with peptides at 95% confidence by MS/MS spectra shown in **Figures 3C, D**. Therefore, decreased ECA binding-galactosylated-CAT and P4HB are selected for further research.

**Table 5:** Major results of the mass spectrometry analysis.

Rank	Unuesd score	UniProtKB number	Abbreviation	Full name	Length (aa)	Molecular weight (kD)
1	28.73	P04040	CAT	Catalase	527	59.756
2	26.48	P07237	P4HB	Protein disulfide-isomerase	508	57.116
3	25.31	P07099	EPHX1	Epoxide hydrolase 1	455	52.949
4	22.9	Q13423	NNT	NAD(P) transhydrogenase, mitochondrial	1,086	113.896
5	22.46	P55084	HADHB	Trifunctional enzyme subunit beta, mitochondrial	474	51.294
6	18.61	P27338	MAOB	Amine oxidase [flavin-containing] B	520	58.763
7	17.47	Q02413	DSG1	Desmoglein-1	1,049	113.748
8	12.27	P31930	UQCRC1	Cytochrome b-c1 complex subunit 1, mitochondrial	480	52.646
9	11.29	P07355	ANXA2	Annexin A2	339	38.604
10	10.63	P11021	HSPA5	Endoplasmic reticulum chaperone BiP	654	72.333
11	9.88	P50440	GATM	Glycine amidinotransferase, mitochondrial	423	48.455
12	9.17	P06576	ATP5F1B	ATP synthase subunit beta, mitochondrial	529	56.56
13	8.61	P27797	CALR	Calreticulin	417	48.142
14	8.4	P07327	ADH1A	Alcohol dehydrogenase 1A	375	39.859
15	7.76	P33121	ACSL1	Long-chain-fatty-acid—CoA ligase 1	698	77.943
16	7.74	P01857	IGHG1	Immunoglobulin heavy constant gamma 1	330	36.106
17	7.35	P22695	UQCRC2	Cytochrome b-c1 complex subunit 2, mitochondrial	453	48.443
18	6.67	P22310	UGT1A4	UDP-glucuronosyltransferase 1-4	534	60.025
19	6.53	P11509	CYP2A6	Cytochrome P450 2A6	494	56.501
20	6.45	Q8NBX0	SCCPDH	Saccharopine dehydrogenase-like oxidoreductase	429	47.151

20 proteins with the highest Unuesd scores. aa: amino acid.

### Much Less Membrane Galactosylated CAT and P4HB Expression in High Metastatic HCC Cells Compared to Low Metastatic and Normal Liver Cells

**Figure 4:**
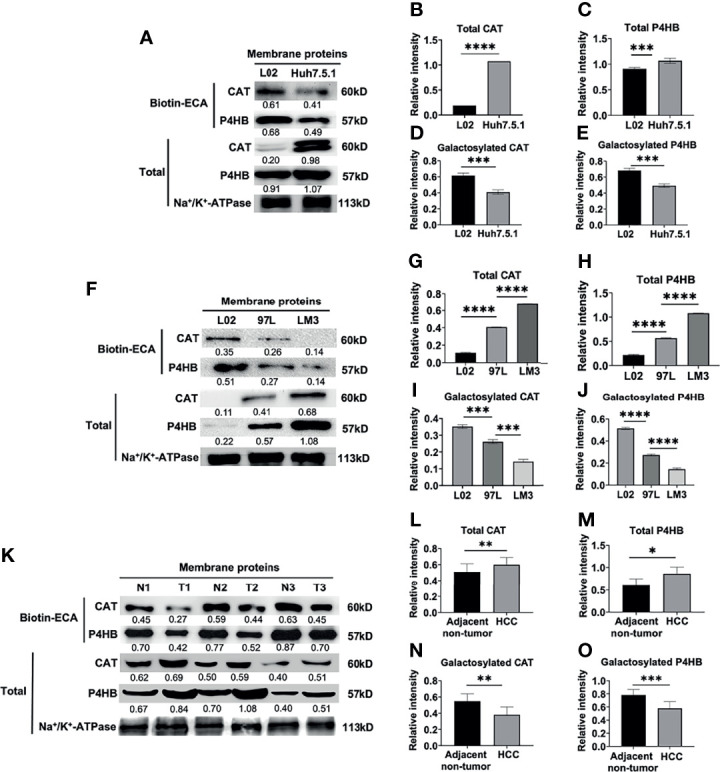
Biotin-conjugated ECA pull down and western blot analysis of decreased membrane glycoproteins CAT and P4HB in different HCC cell lines and HCC tissues. **(A–E)** ECA lectin pull down and western blot analysis of membrane glycoprotein and total CAT and P4HB in human hepatocarcinoma cell line Huh7.5.1 and human normal liver cell L02. **(F–J)** Biotin-conjugated ECA pull down and western blot analysis of membrane glycoprotein and total CAT and P4HB from low metastatic cell (MHCC-97L), high metastatic cell (HCC-LM3) and human normal liver cell L02. **(K–O)** Biotin-conjugated ECA lectin pull down and western blot analysis of membrane glycoprotein and total CAT and P4HB from 3 HCC tissues (T) and adjacent non-tumor tissues **(N)**. P1, P2 and P3 represent HCC tissues from three patients; N1, N2 and N3 represent adjacent-non-tumor tissues from three HCC patients, respectively. **(A, F, K)** are the representative data for **(B–E)**, **(G-J, L-O)**, respectively. **(B, D, I, L, N)** (for CAT), **(C, E, I. J, M, O)** (for P4HB) are statistical analysis. Relative intensities based on endogenous membrane protein Na+/K+-ATPase are presented as the mean ± SD using unpaired Student’s t-tests for **(B–E, L–O)**, and using one way ANOVA followed by Sidak’s multiple comparisons test for **(G–J)** (*p <  0.05, **p < 0.01; ***p < 0.001. ****p < 0.0001).

First, we detected the total membrane CAT and P4HB proteins expression by western blot. A higher expression of total membrane CAT and P4HB was found in the Huh7.5.1 cell line compared to L02 (***p <0.001, **Figures 4A–C** **)**. Next, biotin-conjugated ECA pull-down and western blot assay were performed to verify the expressional changes of ECA-binding-membrane-galactosylated CAT and P4HB proteins in HCC Huh7.5.1 cell line and normal L02 liver cell line. ECA-binding-galactosylated-CAT and P4HB protein expressions were dramatically decreased in the HCC Huh7.5.1 cell line compared to the L02 cell line (***p <0.001, **Figures 4A, D, E** **)**. These results reveal that an increased expression of total CAT and P4HB proteins and a decreased β1,3/β1,4 galactosylated-CAT and P4HB on the cell membrane of the HCC cell line correlate with HCC malignancy.

MHCC-97L is a low metastatic liver cell line, while HCC-LM3 cell is a high metastatic cell line. We examined the total membrane CAT and P4HB protein expression and found a relatively higher expression of total CAT and P4HB proteins in the normal L02 liver cell line compared to the low metastatic MHCC-97L and high metastatic HCC-LM3 cell line (***p <0.001, **Figures 4F–H** **).** However, a progressively decreased expression of ECA-binding-galactosylated-CAT and P4HB was found in the high metastatic HCC-LM3 cell line compared to the low metastatic MHCC-97L and normal L02 liver cell line (***p <0.001, **Figures 4F, I, J** **)**. These results indicated that increased expression of total membrane CAT and P4HB proteins and decreased galactosylated membrane CAT and P4HB proteins may be correlated with HCC malignant metastasis.

Consistent with the results from HCC cell lines, the total membrane CAT and P4HB expression were higher in HCC tissues compared with adjacent non-tumor tissues (**Figures 4K–M** **)**. We further measured the expression levels of ECA-binding-galactosylated-CAT and P4HB on the cellular membrane of HCC tissues vs. adjacent non-tumor tissues by using biotin-conjugated ECA pull-down and western blot analysis as shown in **Figures 4K, N, O**. We also found much lower expression levels of ECA-binding-membrane CAT and P4HB in HCC tumor tissues than adjacent non-tumor tissues from three HCC patients (**Figures 4K, N, O** **)**. All the above results reveal a remarkably higher total membrane CAT and P4HB proteins and a significantly lower galactosylated membrane CAT and P4HB proteins expression in both HCC cell lines and HCC tissues compared with normal liver cells and adjacent non-tumor tissues.

## Discussion

Most tumor biomarkers are glycoproteins. In view of the complex relationship between the diversity of glycans and tumors, and the importance of glycosylation in cell migration, proliferation, and differentiation, proteins with specific glycans have been used as valuable diagnostic, prognostic, and therapeutic biomarkers in liver cancer, breast cancer, lung cancer, and other malignant tumors and can further serve as important targets for tumor diagnosis and therapy (26). The high-throughput glycoproteomics technology based on the lectin array for screening tumor biomarkers has been recently widely used in the clinical research of malignant tumors (27). Lectins are biomolecules found in nature with specific affinities toward particular glycan structures, thus forming a relatively strong complex (28, 29). Because of this characteristic, lectins have been used in analytical techniques (e.g., lectin affinity chromatography) for the selective capture or separation of certain glycans in complex samples, or used in lectin microarrays for characterizing glycosylation profiles in diverse clinical situations (30). Lectins have also been developed for the detection of specific aberrant and cancer-associated glycostructures to assist diagnosis, prognosis based on the assessment of patient serum glycoproteins using lectins, such as Sialic acid-binding immunoglobulin-like lectin 15 (Siglec-15) (31), Galectin-8 (32), and Galectin-3 (33).

The lectin ECA was isolated from the *E. cristagalli* seeds, which can specifically recognize and bind with Gal (galactose) β1-3GlcNAc (N-acetylglucosamine)/Galβ1-4GlcNAc glycans (25). From our results, we showed that ECA had a good diagnostic performance with a statistically significant difference and a high AUC value with a sensitivity of 85% and specificity of 75%, which can efficiently differentiate HCC tissues from adjacent non-tumor tissues. We also showed that the combined application of ECA and serum AFP acquired a much higher specificity at 90% and sensitivity at 85% than ECA or AFP assay alone. To our knowledge, this is the first report showing that abnormal declined β1,3/β1,4 galactosylated membrane glycans-binding ECA can serve as a potential biomarker for HCC diagnosis or malignant prognosis.

In the present study, we also firstly found and determined two abnormal declined membrane galactosylated-CAT and P4HB glycoproteins as potential biomarkers in HCC diagnosis and malignancy progression prediction. CAT is a key enzyme in the metabolism of H_2_O_2_ and reactive oxygen species (ROS) (34). Previous studies have shown that the functional CAT is mainly located in peroxisomes; moreover, it has also been found in the cytoplasm, mitochondria, and on the cytoplasmic membrane of human cancer cells (35). So far, elevated expression of total CAT protein level has been found on the cell surface of tumor cells such as gastric cancer, skin cancer, colon cancer, and chronic myeloid leukemia (36–39). Consistently, in this study, we also found an increased total CAT expression, and we also firstly found that there was significantly decreased ECA-binding membrane galactosylated CAT of human HCC tissues compared to adjacent non-tumor tissues, and decreased as the malignancy of liver cancer increased accordingly, which suggest that both increased total CAT protein and decreased galactosylated CAT glycoprotein level might be involved in the development of HCC.

P4HB is a multifunctional protein that catalyzes the formation and rearrangement of disulfide bonds. It can act as a molecular chaperone to refine misfolded proteins in response to endoplasmic reticulum (ER) stress (40). It has been reported that P4HB is indicated as a diagnosis and prognosis biomarker and the abnormal higher expression of total P4HB protein level has been found in various tumor types, such as renal cell carcinoma (41), bladder carcinoma (24), gastric cancer (42), diffuse gliomas (43), lung cancer (44), and hepatocellular carcinoma (45). The high P4HB was associated with HCC tumorigenesis and epithelial-to-mesenchymal transition (45, 46). Consistently, in this study, we also found an increased total P4HB expression, and we also firstly identified that membrane galactosylated modification of P4HB in HCC tissues was reduced compared with adjacent non-tumor tissues, and decreased galactosylated-P4HB was associated with HCC malignancy and metastasis. These data clearly suggest that both increased total P4HB and decreased galactosylated P4HB might be involved in the development of HCC.

Our analysis still has some limitations. First, a larger sample size and multicenter study might be needed to further confirm our results. Second, the N/O-glycan profile characteristic of ECA binding galactosylated membrane glycoproteins CAT and P4HB needs further elucidation. Third, the regulation mechanism of decreased galactosylated membrane glycoproteins CAT and P4HB in HCC needs to be further explored. But our study provided comprehensive information of HCC-associated cellular membrane glycopatterns and ECA/ECA-binding membrane CAT and P4HB glycoproteins that may contribute to understanding the complex physiological changes of HCC patients. Our results also provided a new insight for the research of HCC biomarkers and anti-tumor drug targets by using ECA-binding membrane CAT and P4HB glycoproteins, which is conducive to understanding HCC mechanisms and provides a set of potential targets for diagnostic application and therapeutic strategies.

## Data Availability Statement

The original contributions presented in the study are included in the article/supplementary material. Further inquiries can be directed to the corresponding author.

## Ethics Statement

The studies involving human participants were reviewed and approved by the Ethical Committee of Wuhan University School of Medicine. The patients/participants provided their written informed consent to participate in this study.

## Author Contributions

YK, HC, MC, YL, JL and QL performed the experiments and analyzed the data. TG and YY provided the test samples. TG, YY, and YX assisted in the analysis of clinical data. HX contributed IHC stain expertise. XLZ initiated the study, analyzed data, and created and revised the manuscript. All authors listed have made a substantial, direct, and intellectual contribution to the work and approved it for publication.

## Funding

This work was supported by grants from the National Key R&D Program of China (2018YFA0507603), the National Natural Science Foundation of China (91740120, 22077097, 21721005, and 21572173), the National Grand Program on Key Infectious Disease of China (2017ZX10201301-006), the National Outstanding Youth Foundation of China (81025008), the Medical Science Advancement Program (Basic Medical Sciences) of Wuhan University (TFJC 2018002), the Key R&D Program of Hubei Province (2020BCB020), the Hubei Province’s Outstanding Medical Academic Leader Program (523-276003), the Innovative Group Project of Hubei Health Committee (WJ2021C002), and the Foundational Research Funds for the Central University of China.

## Conflict of Interest

The authors declare that the research was conducted in the absence of any commercial or financial relationships that could be construed as a potential conflict of interest.

## Publisher’s Note

All claims expressed in this article are solely those of the authors and do not necessarily represent those of their affiliated organizations, or those of the publisher, the editors and the reviewers. Any product that may be evaluated in this article, or claim that may be made by its manufacturer, is not guaranteed or endorsed by the publisher.

## References

[1] Zheng R Qu C Zhang S Zeng H Sun K Gu X. Liver Cancer Incidence and Mortality in China: Temporal Trends and Projections to 2030. Chin J Cancer Res (2018) 30:571–9. doi: 10.21147/j.issn.1000-9604.2018.06.01 PMC6328503 30700925

[2] European Association for the Study of the Liver. EASL Clinical Practice Guidelines: Management of Hepatocellular Carcinoma. J Hepatol (2018) 69:182–236. doi: 10.1016/j.jhep.2018.03.019 29628281

[3] Global Burden of Disease Liver Cancer Collaboration. The Burden of Primary Liver Cancer and Underlying Etiologies From 1990 to 2015 at the Global, Regional, and National Level: Results From the Global Burden of Disease Study 2015. JAMA Oncol (2017) 3:1683–91. doi: 10.1001/jamaoncol.2017.3055 PMC5824275 28983565

[4] Befeler AS Di Bisceglie AM. Hepatocellular Carcinoma: Diagnosis and Treatment. Gastroenterology (2002) 122:1609–19. doi: 10.1053/gast.2002.33411 12016426

[5] Gower E Estes C Blach S Razavi-Shearer K Razavi H. Global Epidemiology and Genotype Distribution of the Hepatitis C Virus Infection. J Hepatol (2014) 61:S45–57. doi: 10.1016/j.jhep.2014.07.027 25086286

[6] Gao Q Zhu H Dong L Shi W Chen R Song Z. Integrated Proteogenomic Characterization of HBV-Related Hepatocellular Carcinoma. Cell (2019) 179:561–77.e22. doi: 10.1016/j.cell.2019.08.052 31585088

[7] Satomaa T Heiskanen A Leonardsson I Angstrom J Olonen A Blomqvist M. Analysis of the Human Cancer Glycome Identifies a Novel Group of Tumor-Associated N-Acetylglucosamine Glycan Antigens. Cancer Res (2009) 69:5811–9. doi: 10.1158/0008-5472.CAN-08-0289 19584298

[8] Islami F Miller KD Siegel RL Fedewa SA Ward EM Jemal A. Disparities in Liver Cancer Occurrence in the United States by Race/Ethnicity and State. CA Cancer J Clin (2017) 67:273–89. doi: 10.3322/caac.21402 28586094

[9] Craig AJ von Felden J Garcia-Lezana T Sarcognato S Villanueva A. Tumour Evolution in Hepatocellular Carcinoma. Nat Rev Gastroenterol Hepatol (2020) 17:139–52. doi: 10.1038/s41575-019-0229-4 31792430

[10] Tsuchiya N Sawada Y Endo I Saito K Uemura Y Nakatsura T. Biomarkers for the Early Diagnosis of Hepatocellular Carcinoma. World J Gastroenterol (2015) 21:10573–83. doi: 10.3748/wjg.v21.i37.10573 PMC4588079 26457017

[11] Cao L Cheng H Jiang Q Li H Wu Z. APEX1 Is a Novel Diagnostic and Prognostic Biomarker for Hepatocellular Carcinoma. Aging (2020) 12:4573–91. doi: 10.18632/aging.102913 PMC7093175 32167932

[12] Zhou F Shang W Yu X Tian J. Glypican-3: A Promising Biomarker for Hepatocellular Carcinoma Diagnosis and Treatment. Med Res Rev (2018) 38:741–67. doi: 10.1002/med.21455 28621802

[13] Jiang K Shang S Li W Guo K Qin X Zhang S. Multiple Lectin Assays for Detecting Glyco-Alteration of Serum GP73 in Liver Diseases. Glycoconj J (2015) 32:657–64. doi: 10.1007/s10719-015-9614-6 26342810

[14] Chen L Li M Li Q Wang C-j Xie S-q. DKK1 Promotes Hepatocellular Carcinoma Cell Migration and Invasion Through β-Catenin/MMP7 Signaling Pathway. Mol Cancer (2013) 12:157. doi: 10.1186/1476-4598-12-157 24325363 PMC4029244

[15] Li J Cheng Y Guo J He J Jiang Z Liang J. Guidelines of Chinese Society of Clinical Oncology (CSCO) Hepatocellular Carcinoma Vol. 98. China: People’s Medical Publishing House (2018).

[16] Zalba S ten Hagen TLM. Cell Membrane Modulation as Adjuvant in Cancer Therapy. Cancer Treat Rev (2017) 52:48–57. doi: 10.1016/j.ctrv.2016.10.008 27889637 PMC5195909

[17] Han Y Xiao K Tian Z. Comparative Glycomics Study of Cell-Surface N-Glycomes of HepG2 Versus LO2 Cell Lines. J Proteome Res (2019) 18:372–9. doi: 10.1021/acs.jproteome.8b00655 30343578

[18] Hirabayashi J Yamada M Kuno A Tateno H. Lectin Microarrays: Concept, Principle and Applications. Chem Soc Rev (2013) 42:4443–58. doi: 10.1039/c3cs35419a 23443201

[19] Badr HA AlSadek DMM Darwish AA ElSayed AI Bekmanov BO Khussainova EM. Lectin Approaches for Glycoproteomics in FDA-Approved Cancer Biomarkers. Expert Rev Proteomics (2014) 11:227–36. doi: 10.1586/14789450.2014.897611 24611567

[20] Xiang T Yang G Liu X Zhou Y Fu Z Lu F. Alteration of N-Glycan Expression Profile and Glycan Pattern of Glycoproteins in Human Hepatoma Cells After HCV Infection. Biochim Biophys Acta Gen Subj (2017) 1861:1036–45. doi: 10.1016/j.bbagen.2017.02.014 28229927

[21] Li S Liu XY Pan Q Wu J Liu ZH Wang Y. Hepatitis C Virus-Induced FUT8 Causes 5-FU Drug Resistance in Human Hepatoma Huh7.5.1 Cells. Viruses (2019) 11:378. doi: 10.3390/v11040378 PMC6521249 31022917

[22] Chen H Shi Y Sun L Ni S. Electrospun Composite Nanofibers With All-Trans Retinoic Acid and MWCNTs-OH Against Cancer Stem Cells. Life Sci (2020) 258:118152. doi: 10.1016/j.lfs.2020.118152 32735881

[23] Mazières J Brugger W Cappuzzo F Middel P Frosch A Bara I. Evaluation of EGFR Protein Expression by Immunohistochemistry Using H-Score and the Magnification Rule: Re-Analysis of the SATURN Study. Lung Cancer (2013) 82:231–7. doi: 10.1016/j.lungcan.2013.07.016 23972450

[24] Wu Y Peng Y Guan B He A Yang K He S. P4HB: A Novel Diagnostic and Prognostic Biomarker for Bladder Carcinoma. Oncol Lett (2021) 21:95. doi: 10.3892/ol.2020.12356 33376528 PMC7751343

[25] Wu AM Wu JH Tsai MS Yang Z Sharon N Herp A. Differential Affinities of Erythrina Cristagalli Lectin (ECL) Toward Monosaccharides and Polyvalent Mammalian Structural Units. Glycoconj J (2007) 24:591–604. doi: 10.1007/s10719-007-9063-y 17805962

[26] Wang J Zhou C Zhang W Yao J Lu H Dong Q. An Integrative Strategy for Quantitative Analysis of the N-Glycoproteome in Complex Biological Samples. Proteome Sci (2014) 12:4. doi: 10.1186/1477-5956-12-4 24428921 PMC3923275

[27] Silva MLS. Lectin Biosensors in Cancer Glycan Biomarker Detection. Adv Clin Chem (2019) 93:1–61. doi: 10.1016/bs.acc.2019.07.001 31655728

[28] Silva MLS. Lectin-Based Biosensors as Analytical Tools for Clinical Oncology. Cancer Lett (2018) 436:63–74. doi: 10.1016/j.canlet.2018.08.005 30125611

[29] Yang J Liu X Shu J Hou Y Chen M Yu H. Abnormal Galactosylated-Glycans Recognized by Bandeiraea Simplicifolia Lectin I in Saliva of Patients With Breast Cancer. Glycoconj J (2020) 37:373–94. doi: 10.1007/s10719-020-09910-6 32103424

[30] Yu H Shu J Li Z. Lectin Microarrays for Glycoproteomics: An Overview of Their Use and Potential. Expert Rev Proteomics (2020) 17:27–39. doi: 10.1080/14789450.2020.1720512 31971038

[31] Li B Zhang B Wang X Zeng Z Huang Z Zhang L. Expression Signature, Prognosis Value, and Immune Characteristics of Siglec-15 Identified by Pan-Cancer Analysis. Oncoimmunology (2020) 9:1807291. doi: 10.1080/2162402X.2020.1807291 32939323 PMC7480813

[32] Elola MT Ferragut F Cardenas Delgado VM Nugnes LG Gentilini L Laderach D. Expression, Localization and Function of Galectin-8, a Tandem-Repeat Lectin, in Human Tumors. Histol Histopathol (2014) 29:1093–105. doi: 10.14670/HH-29.1093 24696431

[33] Dong R Zhang M Hu Q Zheng S Soh A Zheng Y. Galectin-3 as a Novel Biomarker for Disease Diagnosis and a Target for Therapy (Review). Int J Mol Med (2018) 41:599–614. doi: 10.3892/ijmm.2017.3311 29207027 PMC5752178

[34] Glorieux C Zamocky M Sandoval JM Verrax J Calderon PB. Regulation of Catalase Expression in Healthy and Cancerous Cells. Free Radic Biol Med (2015) 87:84–97. doi: 10.1016/j.freeradbiomed.2015.06.017 26117330

[35] Heinzelmann S Bauer G. Multiple Protective Functions of Catalase Against Intercellular Apoptosis-Inducing ROS Signaling of Human Tumor Cells. Biol Chem (2010) 391:675–93. doi: 10.1515/bc.2010.068 20370323

[36] Hwang TS Choi HK Han HS. Differential Expression of Manganese Superoxide Dismutase, Copper/Zinc Superoxide Dismutase, and Catalase in Gastric Adenocarcinoma and Normal Gastric Mucosa. Eur J Surg Oncol (2007) 33:474–9. doi: 10.1016/j.ejso.2006.10.024 17129702

[37] Sander CS Hamm F Elsner P Thiele JJ. Oxidative Stress in Malignant Melanoma and Non-Melanoma Skin Cancer. Br J Dermatol (2003) 148:913–22. doi: 10.1046/j.1365-2133.2003.05303.x 12786821

[38] Rainis T Maor I Lanir A Shnizer S Lavy A. Enhanced Oxidative Stress and Leucocyte Activation in Neoplastic Tissues of the Colon. Dig Dis Sci (2007) 52:526–30. doi: 10.1007/s10620-006-9177-2 17195121

[39] Zelen I Djurdjevic P Popovic S Stojanovic M Jakovljevic V Radivojevic S. Antioxidant Enzymes Activities and Plasma Levels of Oxidative Stress Markers in B-Chronic Lymphocytic Leukemia Patients. J BUON (2010) 15:330–6. 20658731

[40] Jang I Pottekat A Poothong J Yong J Lagunas-Acosta J Charbono A. PDIA1/P4HB Is Required for Efficient Proinsulin Maturation and ß Cell Health in Response to Diet Induced Obesity. eLife (2019) 8:e44528. doi: 10.7554/eLife.44528 31184304 PMC6559792

[41] Xie L Li H Ma LZ Dang Y Guo J Liu J. Autophagy-Related Gene P4HB: A Novel Diagnosis and Prognosis Marker for Kidney Renal Clear Cell Carcinoma. Aging (2020) 12:1828–42. doi: 10.18632/aging.102715 PMC7053637 32003756

[42] Zhang J Guo S Wu Y Zheng ZC Wang Y Zhao Y. P4HB, a Novel Hypoxia Target Gene Related to Gastric Cancer Invasion and Metastasis. BioMed Res Int (2019) 2019:9749751. doi: 10.1155/2019/9749751 31467922 PMC6699373

[43] Zou H Wen C Peng Z Shao Y Hu L Li S. P4HB and PDIA3 Are Associated With Tumor Progression and Therapeutic Outcome of Diffuse Gliomas. Oncol Rep (2018) 39:501–10. doi: 10.3892/or.2017.6134 PMC5783617 29207176

[44] Ercan H Mauracher L-M Grilz E Hell L Hellinger R Schmid J. Alterations of the Platelet Proteome in Lung Cancer: Accelerated F13A1 and ER Processing as New Actors in Hypercoagulability. Cancers (2021) 13:2260. doi: 10.3390/cancers13092260 34066760 PMC8125802

[45] Dai J Jiang L Qiu L Shao Y Shi P Li J. WHSC1 Promotes Cell Proliferation, Migration, and Invasion in Hepatocellular Carcinoma by Activating Mtorc1 Signaling. Onco Targets Ther (2020) 13:7033–44. doi: 10.2147/OTT.S248570 PMC7398890 32801739

[46] Xia W Zhuang 1J Wang G Ni J Wang J Ye Y. P4HB Promotes HCC Tumorigenesis Through Downregulation of GRP78 and Subsequent Upregulation of Epithelial-to- Mesenchymal Transition. Oncotarget (2016) 8:8512–21. doi: 10.18632/oncotarget.14337 PMC5352418 28052026

